# Supplementary material for: Harnessing Artificial Intelligence to Enhance Global Breast Cancer Care: A Scoping Review of Applications, Outcomes, and Challenges
Source: Cancers (Basel). 2025 Jan 9;17(2):197. doi: 10.3390/cancers17020197 (PMC11764353; doi:10.3390/cancers17020197)
Supplement: Supplementary file 1 [file cancers-17-00197-s001.zip › cancers-3351564-supplementary.pdf]

Supplementary Table S1: Detailed Search Strategy

Database	Search Strategy
PubMed	(“Artificial Intelligence” OR “Machine Learning” OR “Machine intelligence*” OR “Machine vision*” OR “deep learning” OR “neural network*” OR “robotic*” OR “sentiment analysis”) AND “Breast Cancer” AND (surgery OR “Screen*” OR “diagnosis” OR “treatment”)
Embase	('artificial intelligence':ti,ab,kw OR 'machine learning':ti,ab,kw OR 'machine intelligence':ti,ab,kw OR 'machine vision':ti,ab,kw OR 'deep learning':ti,ab,kw OR 'neural network':ti,ab,kw OR 'robotic':ti,ab,kw OR 'sentiment analysis':ti,ab,kw) AND breast:ti,ab,kw AND cancer:ti,ab,kw AND (surgery:ti,ab,kw OR 'screen':ti,ab,kw OR 'diagnosis':ti,ab,kw OR 'treatment':ti,ab,kw)
Cochrane	((“Artificial Intelligence” OR “Machine Learning” OR “Machine intelligence” OR “Machine vision” OR “deep learning” OR “neural network” OR “robotic” OR “sentiment analysis”)):ti,ab,kw AND (breast cancer):ti,ab,kw AND ((surgery OR “Screen” OR “diagnosis” OR “treatment”)):ti,ab,kw
